# Spirochaetes as intestinal pathogens: Lessons from a *Brachyspira *genome

**DOI:** 10.1186/1757-4749-1-10

**Published:** 2009-05-01

**Authors:** David J Hampson, Niyaz Ahmed

**Affiliations:** 1 Animal Research Institute, School of Veterinary and Biomedical Science, Murdoch University, Murdoch, Western Australia 6150, Australia; 2 Department of Biotechnology, Pathogen Biology Laboratory, School of Life Sciences, University of Hyderabad, Hyderabad 500046, India

## Abstract

Anaerobic spirochaetes of the genus *Brachyspira *have long been known as important gut pathogens of pigs, but increasingly they are recognised as causing disease in birds and other animal species, including human beings. The genome sequence of the major swine pathogen *Brachyspira hyodysenteriae *was recently published, and this revealed extensive genome optimisation that leads to adaptation to the complex environment of the colon. The genome sequences of other pathogenic and non-pathogenic *Brachyspira *species are becoming available, and this data will help to reveal how these species have evolved and adapted to varied lifestyles in the large intestines of different species, and why some but not others can induce colitis and diarrhoea.

## Introduction

Spirochaetes form a distinct monophyletic phylum of bacteria, and contain four genera that contain important pathogenic species, these being *Treponema, Borrelia, Leptospira *and *Brachyspira*. Veterinary microbiologists and clinicians have long recognised the "intestinal spirochaetes" of the genus *Brachyspira *as being important gut pathogens. The best-known species is *Brachyspira hyodysenteriae*, the agent of swine dysentery, which induces an extensive and severe mucohaemorrhagic colitis in growing pigs [1]. There are six other named *Brachyspira *species, with several more species having been unofficially proposed. Of these, *Brachyspira pilosicoli *in particular is now recognised as an important cause of colitis or typhlitis in both pigs and poultry, and it has been suggested that strains of this species are potentially zoonotic [2]. By using appropriate anaerobic culture conditions and/or polymerase chain reaction amplification, these fastidious spirochaetes can be identified in samples from many humans living in crowded or unhygienic conditions in developing countries [3,4]. Recently, the pathogenic potential of *B. pilosicoli *has been emphasised by its identification in the stools of more than one third of cholera patients in Bangladesh, at densities equal to those of *Vibrio cholerae *[5]. Further work is now underway to determine how this spirochaete attaches to colonic enterocytes and induces disease in humans and animals. The first genome sequence of a *Brachyspira *species has provided unprecedented insights into the biology and lifestyle of these pathogens and has opened up a host of new possibilities towards their management in the human and veterinary healthcare arena.

## The genome sequence of *B. hyodysenteriae *– novel insights

Recently the genome of a *Brachyspira *species was sequenced and analysed [6]. In this study *B. hyodysenteriae *strain WA1 was sequenced and subjected to comparative genomic analysis, with a view to improving understanding of how this branch of spirochaete life has adapted to take up residence in the porcine colon, and how it is able to induce such a severe disease in pigs. Surprisingly, of the predicted 2,122 proteins encoded by the genome of approximately 3 Mb, more had similarities to proteins from enteric *Escherichia coli *and *Clostridium *species than they did to proteins of other sequenced spirochaete species. Many of these genes were involved in transport and metabolism functions, which undoubtedly are very important for survival in the dense, complex and changing nutritional and polymicrobial environment of the porcine large intestine. With time, these genes presumably have been acquired by *B. hyodysenteriae *from the other enteric species by horizontal gene transfer, gradually increasing the fitness of the spirochaete to survival in the colonic environment. Why this species preferentially (but not exclusively) colonises the colon of pigs remains unexplained. The very close 16S rRNA gene sequence similarities between the various *Brachyspira *species implies that there has been relatively recent speciation in the genus, but the extent to which new bacterial genes from other enteric species have been incorporated pre- and post-speciation also remains unclear. Further insight into this question will become available as the genome sequences of the other *Brachyspira *species are obtained and subjected to comparative genome analysis, and when further epidemiological studies have helped to reveal the full extent of diversity in the genus.

The potential mechanisms by which *B. hyodysenteriae *may have acquired or exchanged such a broad set of genes were not identified. Two bacteriophage-like genes were present in the genome, as was the full set of genes for the gene transfer agent (GTA) VSH-1, a prophage-like element that is in a state of permanent lysogeny, but is known to be able to transfer random ~7.5 Kb fragments of DNA between *B. hyodysenteriae *strains [7]. Other *Brachyspira *species contain similar GTAs [8], but it is not known whether these allow transfer of DNA across species barriers.

Another apparent adaptation of *B. hyodysenteriae *to a gut lifestyle, that was noted, was the large number of its genes that were associated with chemotaxis and motility. These functions are clearly important in the colonisation process, as, in order to induce disease, the highly motile spirochaete colonises colonic crypts and enters goblet cells, from which it induces a characteristic outpouring of mucus. Other potential virulence factors that were identified included genes predicted to encode 15 proteases and six haemolysins, some of which may be involved in disruption and shedding of colonic enterocytes, exposing the underlying lamina propria to polymicrobial invasion and inflammation. The only genes identified for secretion were those of the common secretory pathway, and no genes encoding known toxin-like proteins were identified. The spirochaete had a full set of genes for lipooligosaccharide (LOS) biosynthesis, but these were not present in a single locus, and surprisingly the *rfb *gene cluster was present on a single ~36 Kb circular plasmid. Previously LOS has been implicated as a potential virulence factor in *B. hyodysenteriae*, inducing local inflammation in the colon.

## Application in vaccine development and epidemiology

The availability of the genome sequence for *B. hyodysenteriae *has already opened the door for vaccine development; for example, using the reverse vaccinology approach several potentially protective protein subunits have been identified [9], and further advances in this area will occur in future years. On another front, genome sequence data make it possible to test evolutionary hypotheses based on a core gene-pool. The phylogenetic relatedness of such core genes could then be harnessed to analyse and dissect larger collection of strains and field isolates by multilocus sequence typing (MLST) or analysis of single nucleotide polymorphisms. The genomic analysis of *B. hyodysenteriae *has already provided the basis for development of a MLST scheme for the spirochaete, which, by examining large numbers of strains from different geographical origins, is being used to help uncover the diversity and origins of this pathogenic species [10].

## Perspective

As more genomic data becomes available, and interest increases in intestinal spirochaetes as potential pathogens of humans and other species, this rather neglected field of research is expected to catch up with mainstream infection biology and ecology through the 'omic' platforms. Taking lessons from the first *B. hyodysenteriae *genome, it will be possible for the scientific community to embark upon sequencing of many different human and animal derived isolates of *Brachyspira*. This will not be a difficult task given that newer sequencing platforms have drastically reduced time and cost of whole genome sequencing. Using comparative genomics, it will be possible to gauge the extent of genomic diversity within the *Brachyspira *genus, and the forces that regulate such diversity during their colonisation of the gut and various niches thereof. Also, it will be possible to know what survival advantages are gained by *Brachyspira *species through lateral gene transfer events that seemed to be a dominant evolutionary force in several pathogens [11]. Consequently, functional screens based on genes of especially non-spirochaete origin will be developed, thus leading to fresh insights into adaptation mechanisms in an ecological and evolutionary perspective. More efforts are therefore, clearly needed at all fronts – from broad epidemiological studies of the various species to detailed functional genomics analysis.

## Competing interests

DJH is involved with the development of recombinant vaccines for *B. hyodysenteriae *and other *Brachyspira *species.
